# Supplementary material for: Deep mutational scanning reveals a correlation between degradation and toxicity of thousands of aspartoacylase variants
Source: Nat Commun. 2024 May 13;15:4026. doi: 10.1038/s41467-024-48481-0 (PMC11091098; doi:10.1038/s41467-024-48481-0)
Supplement: Supplementary file 1 — Supplementary Information [file 41467_2024_48481_MOESM1_ESM.pdf]

Deep mutational scanning reveals a correlation between degradation and toxicity of thousands of aspartoacylase variants

Supplementary Information

Supple. Fig. 1, *No detectable endogenous ASPA in HEK293T cells.* p.2
Supple. Fig. 2, *Abundance score correlations between independent repeats.* p.3
Supple. Fig. 3, *Microscopy and solubility of selected variants.* p.4
Supple. Fig. 4, *The* Zn^{2+} *coordinating residues and β-strand at 150-160.* p.5
Supple. Fig. 5, *Many low abundance variants are buried.* p.6
Supple. Fig. 6, *Comparisons of the abundance map with the Rosetta stability predictions.* p.7
Supple. Fig. 7, *Comparisons of the abundance map with the in silico predictions.* p.9
Supple. Fig. 8, *Flow cytometry profiles of the WT and C152W variants.* p.10
Supple. Fig. 9, *Inherent degrons are buried in the ASPA structure.* p.11
Supple. Fig. 10, *Positioning of conserved and catalytic residues.* p.12
Supple. Fig. 11, *The screen for toxic ASPA variants.* p.13
Supple. Fig. 12, *Toxicity score correlations between independent repeats.* p.14
Supple. Fig. 13, *Toxicity score distribution.* p.15
Supple. Fig. 14, *ASPA toxicity scores and abundance determined in low throughput.* p.16
Supple. Fig. 15, *Comparisons of the mutational maps.* p.17
Supple. Fig. 16, *Toxic variants are typically buried.* p.19
Supple. Fig. 17, *Benign variants are not toxic.* p.20
Supple. Fig. 18, *Common variants are not toxic.* p.21
Supple. Fig. 19, *Toxic variants activate a stress response leading to HSP70 induction.* p.22
Supple. Fig. 20, *Flow cytometry gating strategy.* p.23

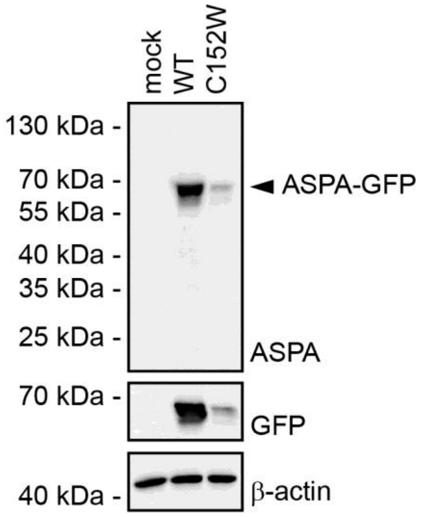

Supplementary Figure 1 – *No detectable endogenous ASPA in HEK293T cells.*
The level of endogenous and recombinant ASPA in the HEK293T landing pad cell line was analyzed was by SDS-PAGE and western blotting using antibodies to ASPA and GFP. β-actin served as a loading control. Note the lack of detectable ASPA signal in the mock sample.

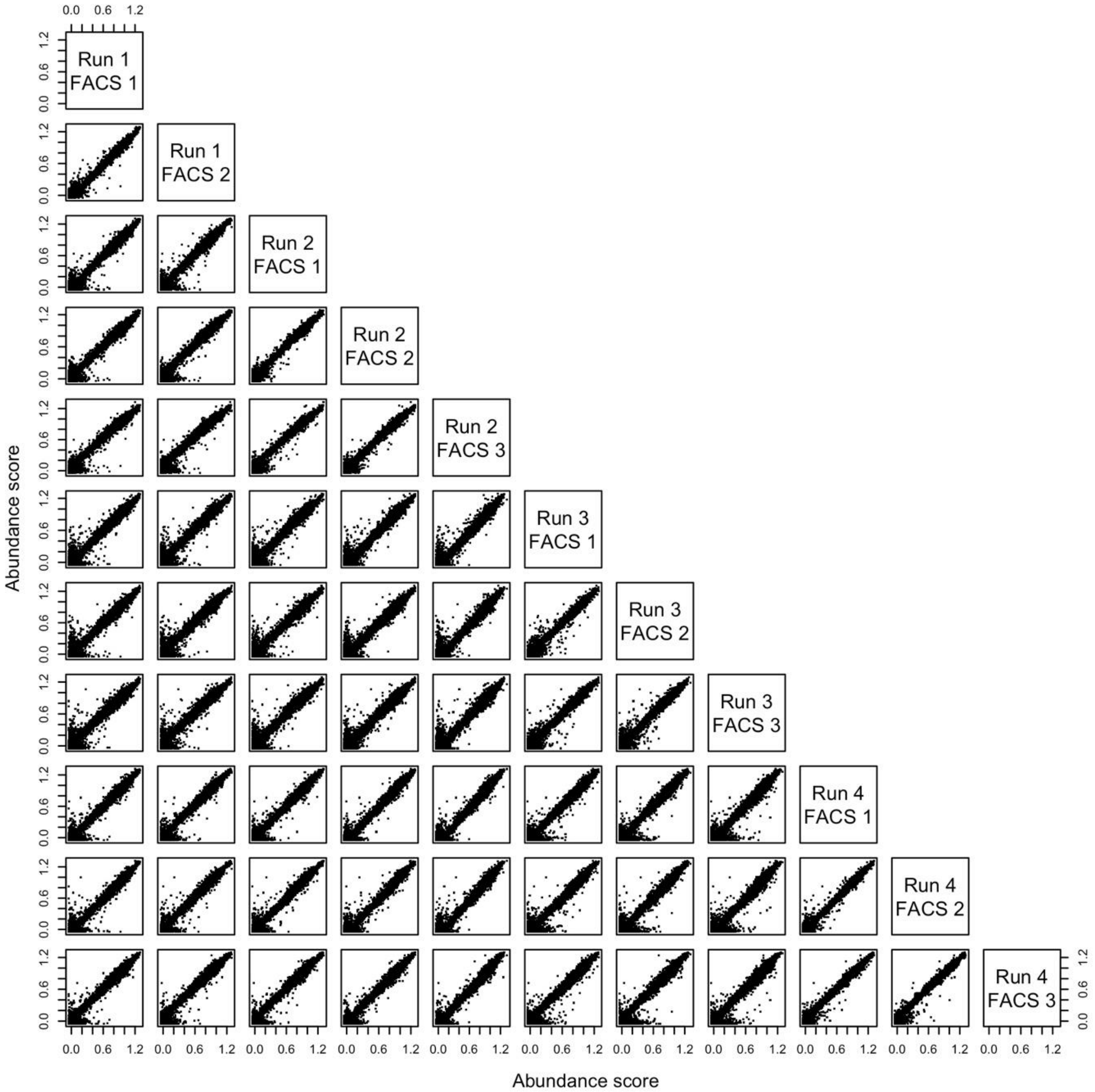

Supplementary Figure 2 – *Abundance score correlations between independent repeats.*
Correlation of single amino acid variant scores between all four biological replicates (Run 1-4) each with three FACS replicates (FACS 1-3) except for B1 which only have two FACS replica (FACS 1-2). All Pearson correlations are in the range 0.98 to 0.99. Note the reduced correlation for the low abundance variants as discussed in the main text.

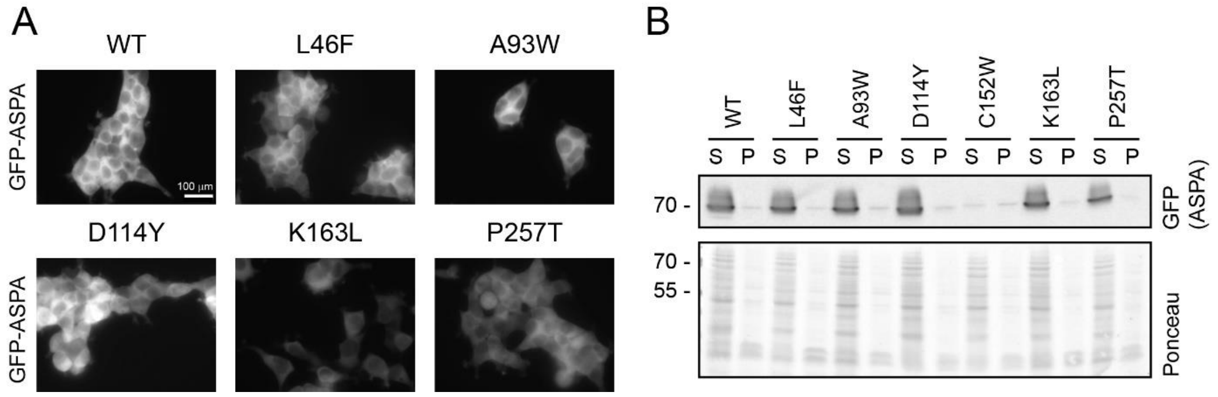

Supplementary Figure 3 – *Microscopy and solubility of selected variants.*
(A) Live cell fluorescence microscopy of HEK293T landing pad cells expressing either wild-type ASPA (WT) or the indicated ASPA variants fused to GFP (as in the main text Fig. 1B). Scale bar = 100 μm. (B) Whole cell lysates of HEK293T cells expressing the indicated ASPA variants from the landing pad were separated into soluble supernatant (S) and insoluble pellet (P) fractions by centrifugation. The fractions were analyzed by SDS-PAGE and western blotting using antibodies to GFP. Ponceau S staining of the membrane serves as a loading control. Note that all variants appear largely in the soluble fractions, while C152W is expressed at a very low level (compare with main text, Fig. 2C) and appears equally distributed between the soluble and insoluble fractions but does not form visible aggregates (main Fig. 1B).

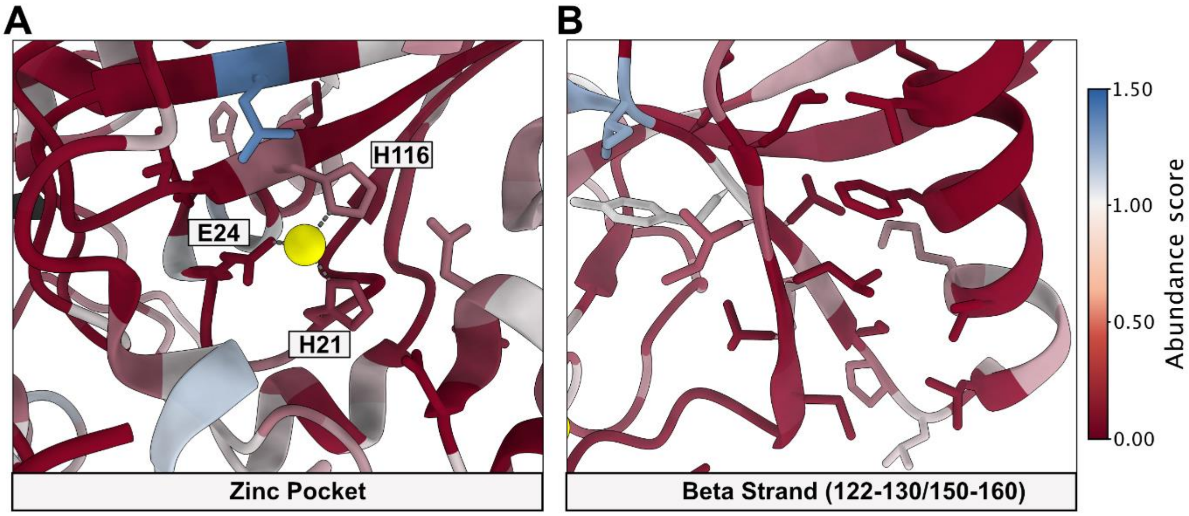

Supplementary Figure 4 – *The Zn^{2+} coordinating residues and β-strand at 150-160.*
(A) The Zn^{2+} coordinating residues in ASPA. The structure is colored based on the median abundance score per residue. Note that the residues in contact with the Zn^{2+} ion are sensitive to substitutions (red). (B) The alternating pattern of low and high abundance variants in the β-strand at position 150-160 is shown. Thus, residues pointing inwards are sensitive to substitutions (red), while residues pointing outwards are tolerant. The structure is colored based on the median abundance score per residue.

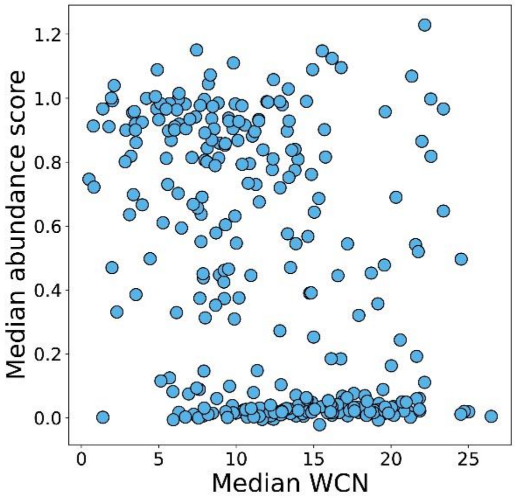

Supplementary Figure 5 – *Many low abundance variants are buried.*
Plot of the median abundance score per residue vs. the weighted contact number (WCN) per position. Positions with a high WCN are buried and appear more sensitive to substitutions (low median abundance score), while exposed positions with a low WCN appear more tolerant to substitutions (high median abundance score).

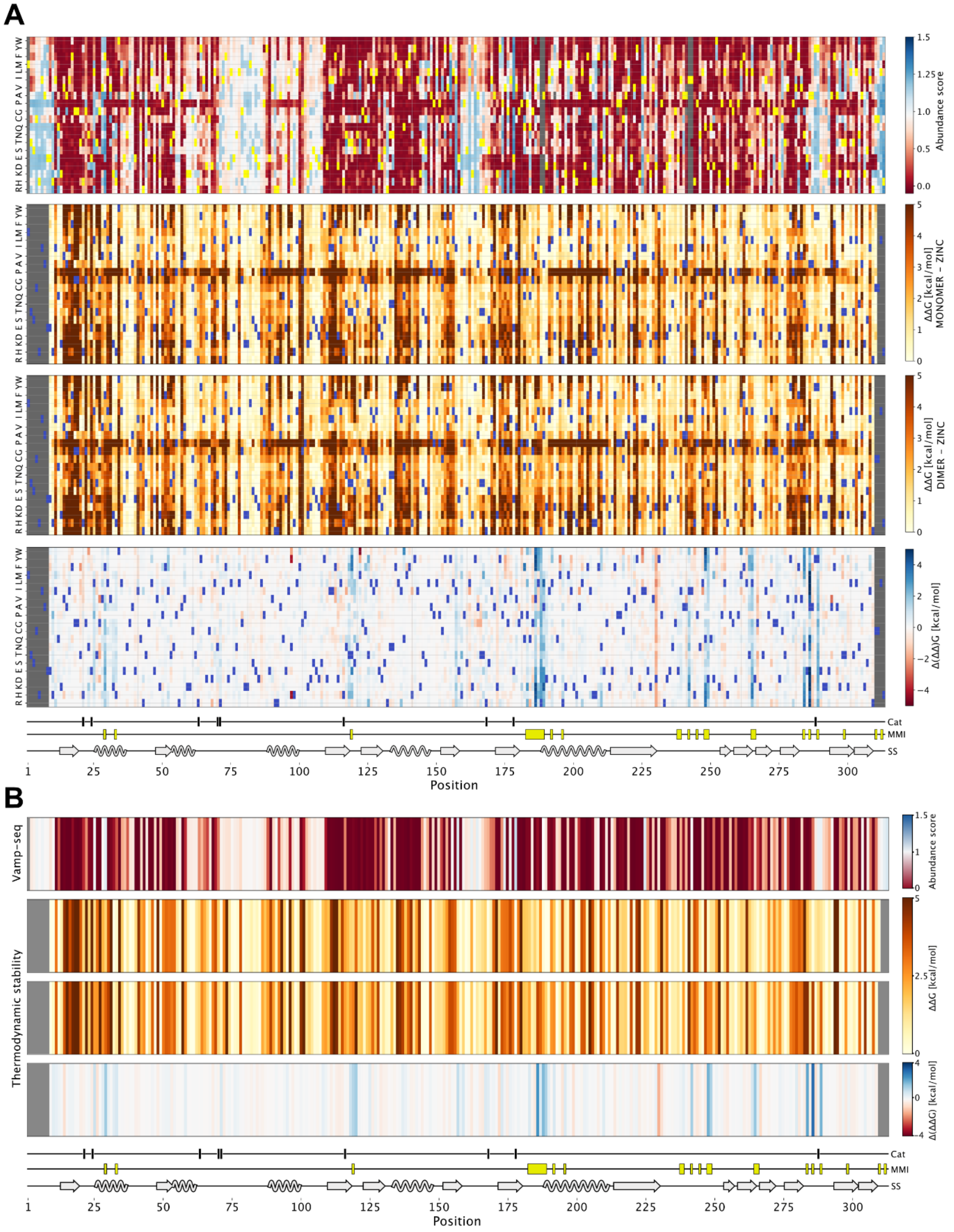

Supplementary Figure 6 – *Comparisons of the abundance map with the Rosetta stability predictions.* (A) Side-by-side comparisons of the ASPA abundance map with the Rosetta stability maps for the ASPA dimer (with zinc) and ASPA monomer (without zinc). The difference in predicted stability (ΔΔΔG) between the ASPA monomer and dimer is also included (lower panel). The secondary structure (SS) elements and monomer-monomer interface (MMI) positions are included for comparison. The position of selected catalytic sites (H21, N23, R63, N70, R71, D114, N117, E178, G185, P232, A287, Y288) is marked (Cat). (B) Comparisons of the ASPA median abundances per residue with the corresponding Rosetta stability predictions for the ASPA dimer and monomer. The difference in

predicted stability ($\Delta\Delta\Delta G$) between the ASPA monomer and dimer is also included (lower panel). The secondary structure (SS) elements and monomer-monomer interface (MMI) positions are included for comparison. The position of selected catalytic sites (H21, N23, R63, N70, R71, D114, N117, E178, G185, P232, A287, Y288) is marked (Cat). Scatter plots of the data are presented in the main paper (Fig. 3A).

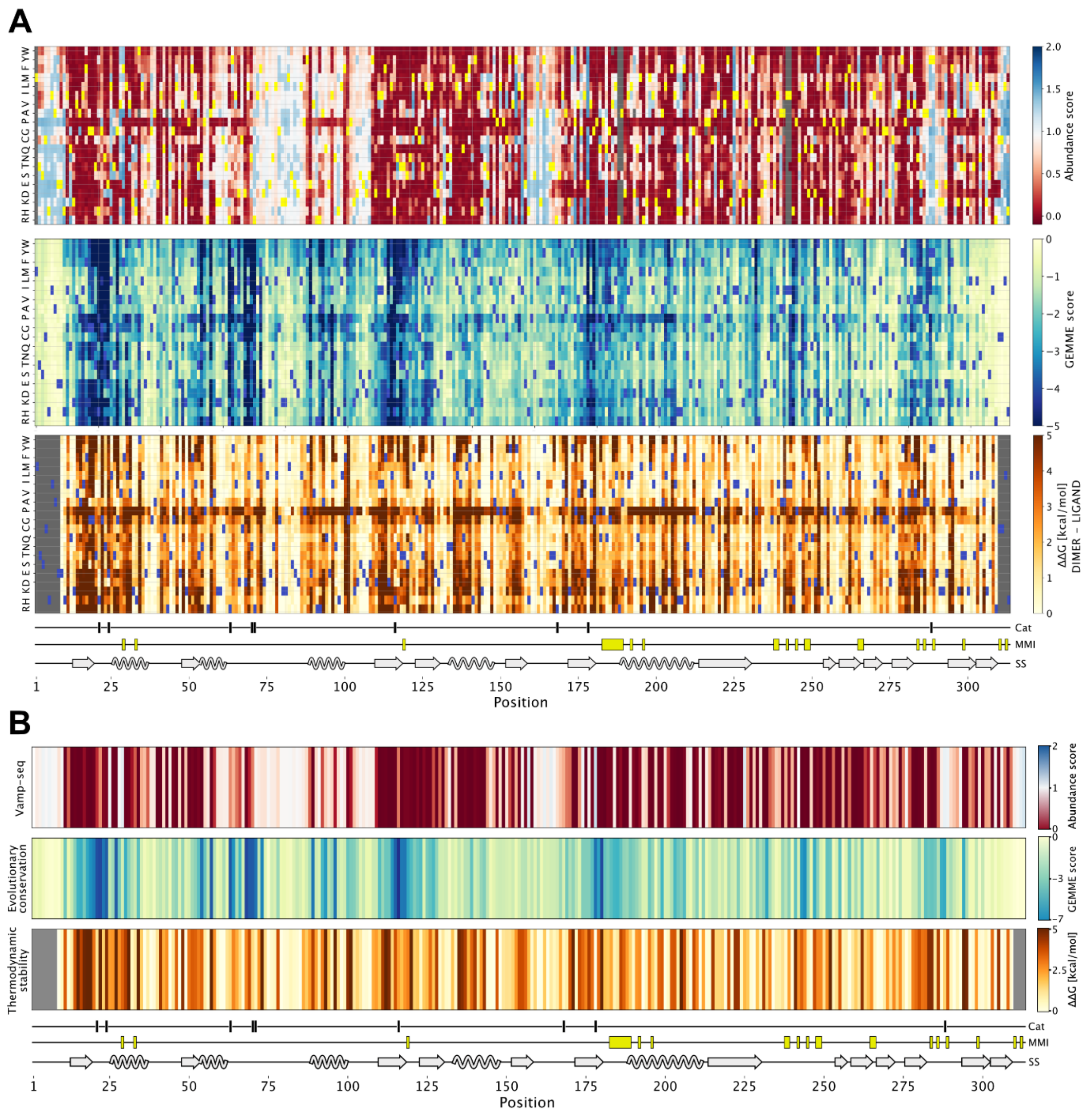

Supplementary Figure 7 – *Comparisons of the abundance map with the in silico predictions.*
(A) Side-by-side comparisons of the ASPA abundance map with the GEMME (ΔΔE) conservation and Rosetta stability maps for the ASPA dimer. The secondary structure (SS) elements and monomer-monomer interface (MMI) positions are included for comparison. The position of selected catalytic sites (H21, N23, R63, N70, R71, D114, N117, E178, G185, P232, A287, Y288) is marked (Cat). (B) Comparisons of the ASPA median abundances per residue with the corresponding residue median GEMME and median Rosetta stability predictions for the ASPA dimer. The secondary structure (SS) elements and monomer-monomer interface (MMI) positions are included for comparison. The position of selected catalytic sites (H21, N23, R63, N70, R71, D114, N117, E178, G185, P232, A287, Y288) is marked (Cat). Scatter plots of the data are presented in the main paper (Fig. 3AB).

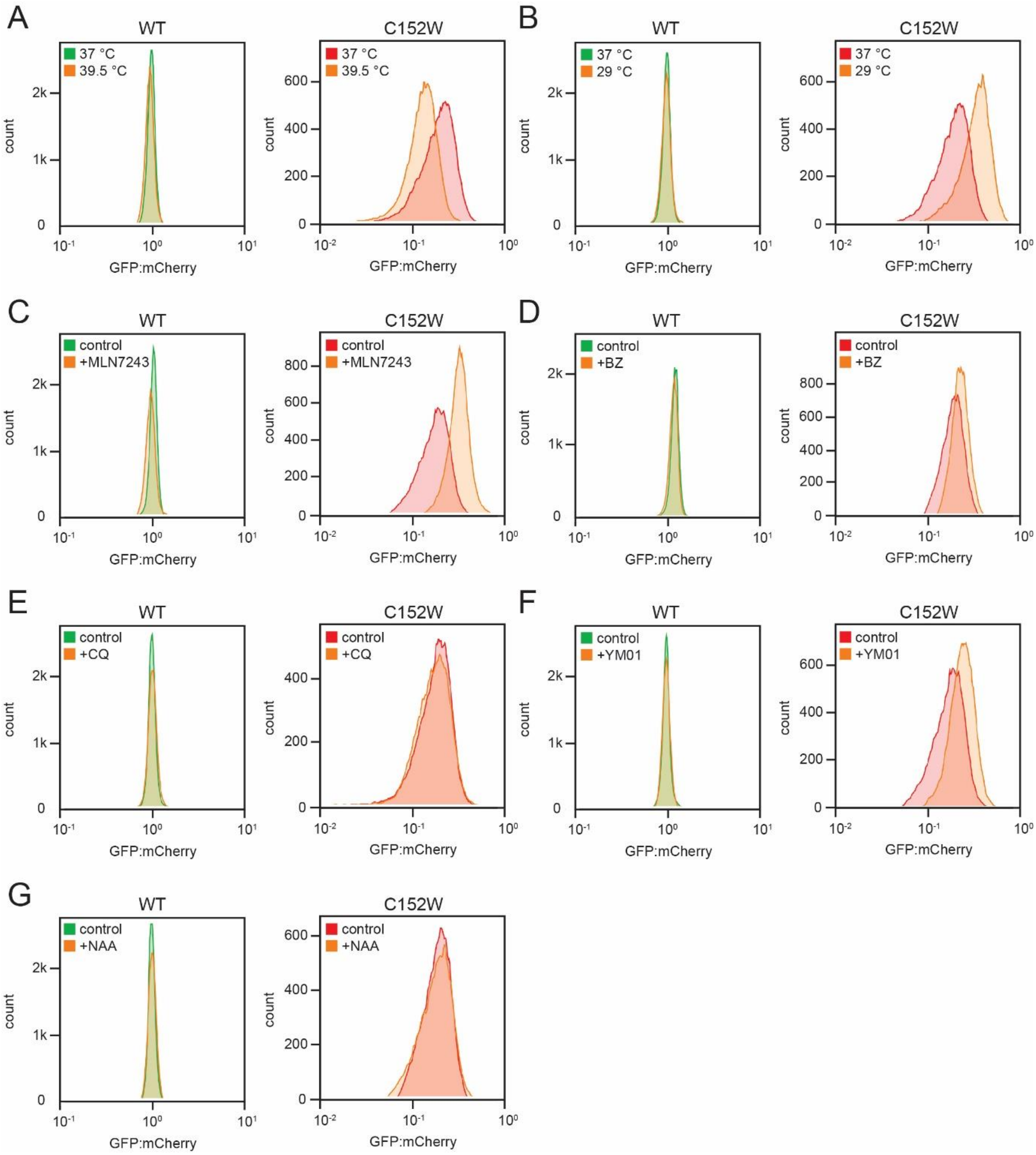

Supplementary Figure 8 – *Flow cytometry profiles of the WT and C152W variants.*
Histograms displaying the distributions of the GFP:mCherry ratios of the WT and C152W ASPA variants as indicated in response to 16 hours of the following treatments prior to harvest and analyses: (A) 39.5 °C, (B) 29 °C, (C) 1 µM of the ubiquitin E1-inhibitor MLN7243, (D) 15 µM of the proteasome inhibitor bortezomib (BZ), (E) 20 µM the lysosomal inhibitor chloroquine (CQ), (F) 2.5 µM of the HSP70-inhibitor YM01 and (G) 6 mM N-acetyl-aspartate (NAA). WT control (green), WT treated (orange), C152W control (red), C152W treated (orange).

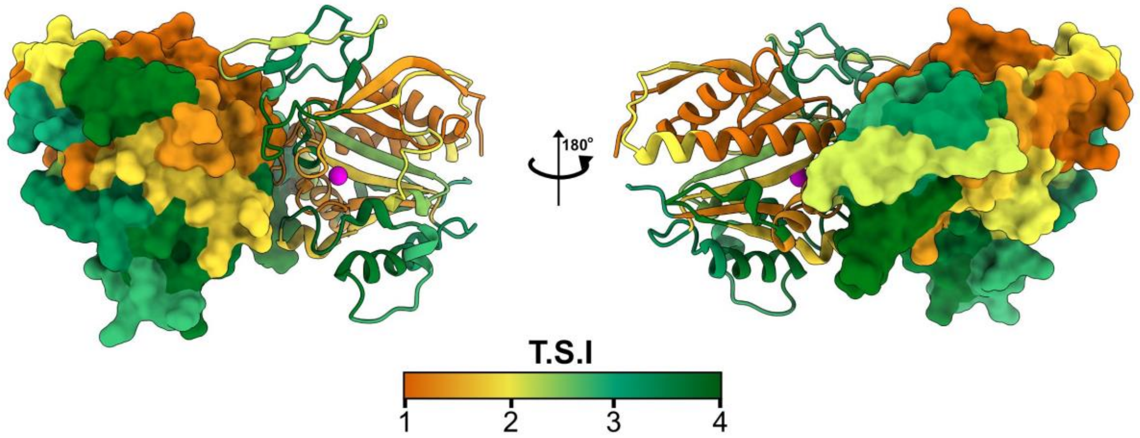

Supplementary Figure 9 – *Inherent degrons are buried in the ASPA structure*.
The ASPA structure colored by tile stability index (TSI). Note that regions with low stability (degrons) are mostly buried in the structure (orange), while stable tiles (green) are exposed.

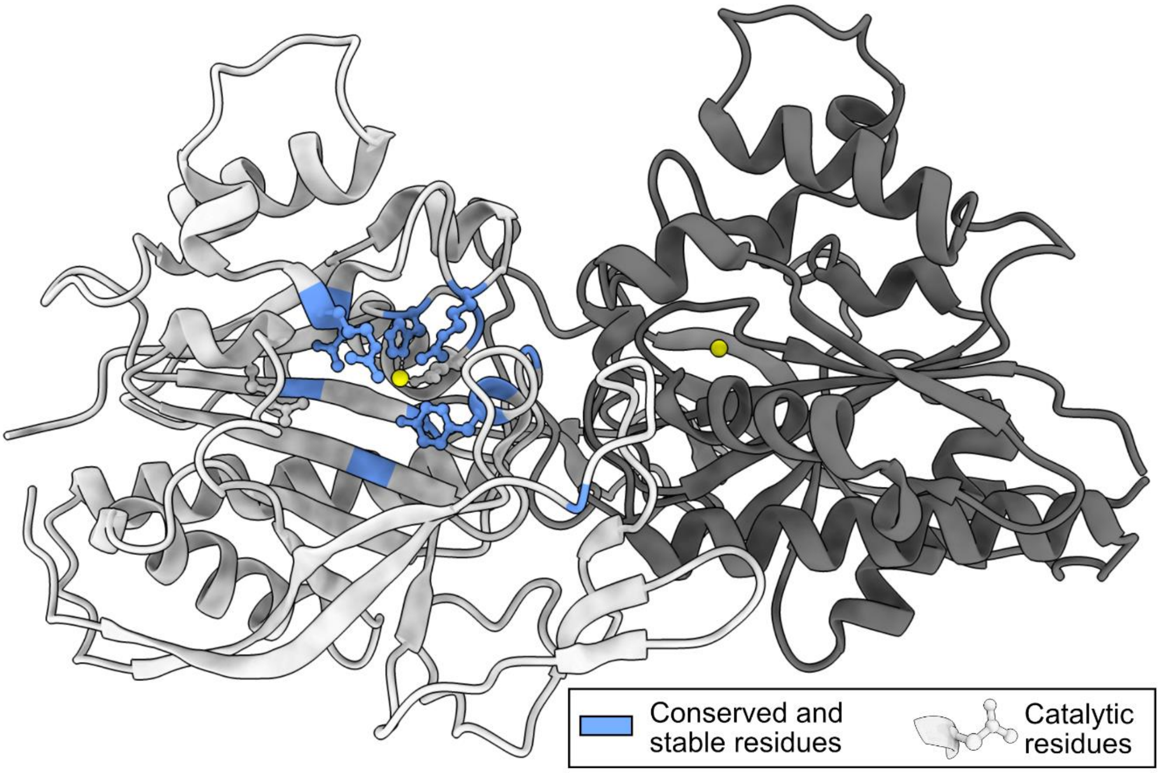

Supplementary Figure 10 – *Positioning of conserved and catalytic residues.*
The ASPA structure with the positions of conserved and stable residues (median Rosetta predicted ΔΔG lower than 2 kcal/mol, and a GEMME evolutionary score lower than -3.5) colored in blue and catalytic residues marked with side-chains.

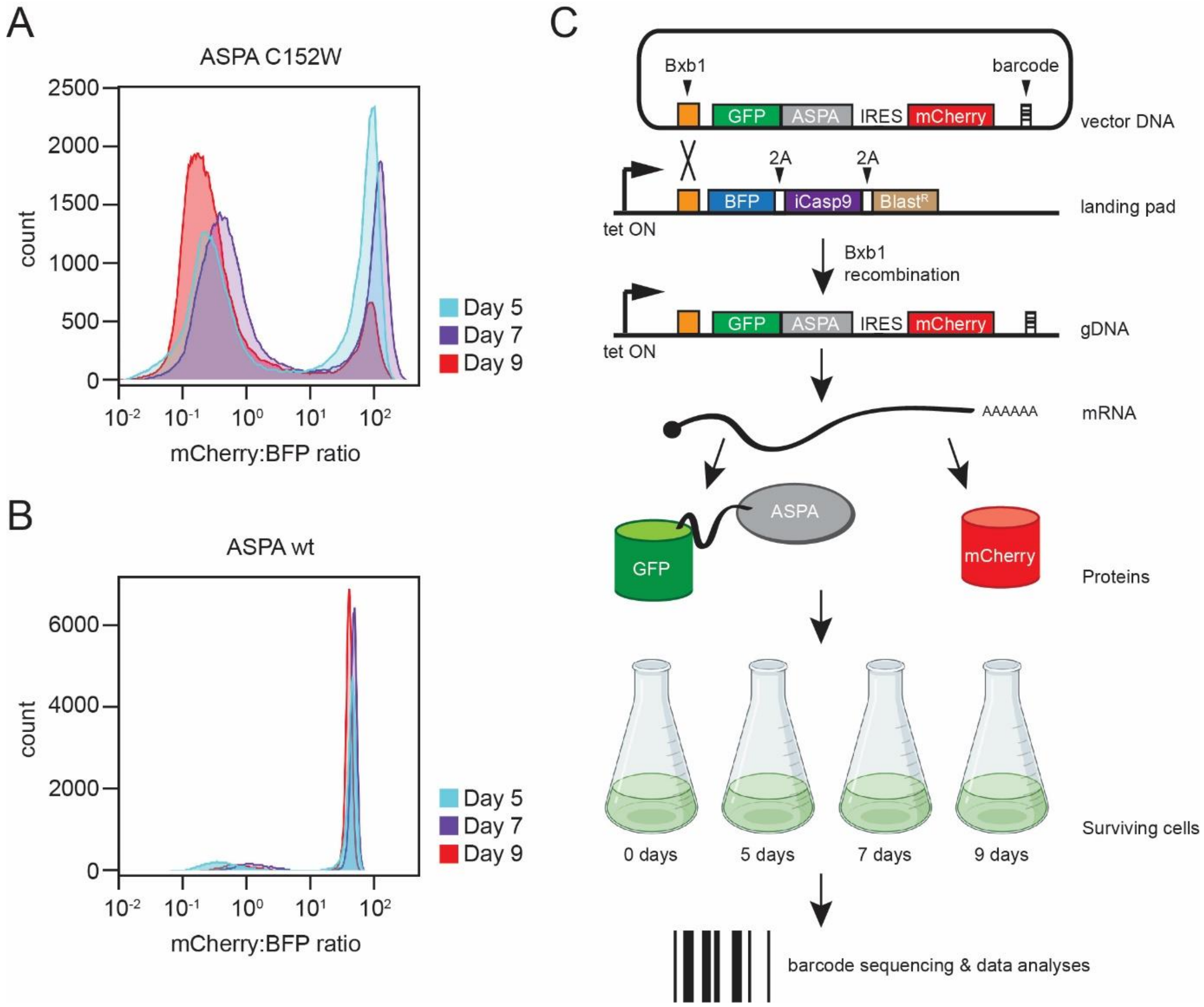

Supplementary Figure 11 – *The screen for toxic ASPA variants.* (A) Histogram of the mCherry:BFP ratio based on flow cytometry ASPA C152W after 5 (cyan), 7 (purple) and 9 (red) days of expression with doxycyclin. Note that over time the number of mCherry-expressing (ASPA C152W positive) cells declines, whereas the number of non-recombined (BFP positive) cells increase. (B) Histogram of the mCherry:BFP ratio based on flow cytometry wild-type ASPA after 5 (cyan), 7 (purple) and 9 (red) days of expression with doxycyclin. (C) Schematic representation of the expression and screening system. HEK293T cells carrying a landing pad for Bxb1-catalyzed site-specific integration are transfected with the expression vector and a Bxb1 expression plasmid (not shown). Upon integration at the landing pad locus, the BFP-iCasp9-Blast^R gene is displaced downstream, and the cells therefore become resistant to AP1903, while GFP-ASPA and mCherry is expressed from the tetracyclin/doxycyclin regulated promoter. The same mRNA leads to both GFP-ASPA and mCherry protein production. Without flow sorting, cells were harvested after 0, 5, 7 and 9 days culturing with doxycyclin. Surviving variants were identified by sequencing the barcodes. Figure adapted from [58,76,96]. Figure created with BioRender.com, released under a Creative Commons Attribution-NonCommercial-NoDerivs 4.0 International license.

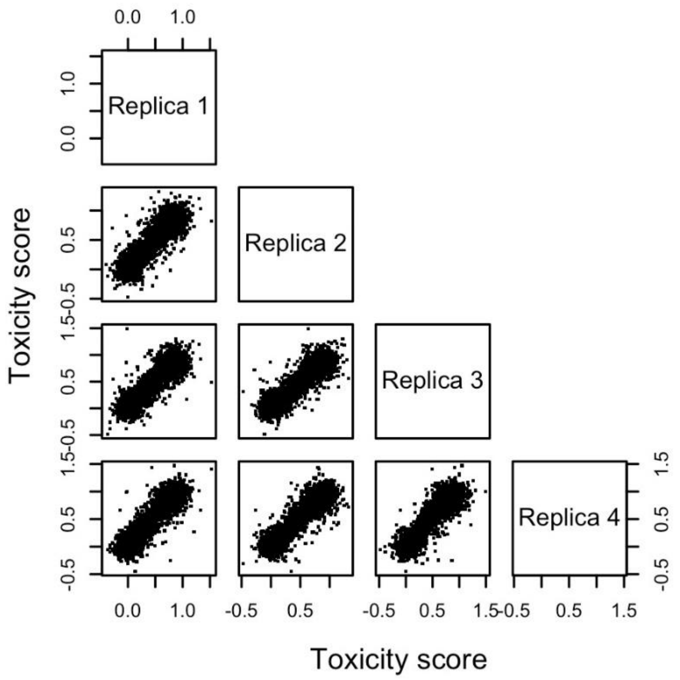

Supplementary Figure 12 – *Toxicity score correlations between independent repeats.*
Correlation of single amino acid variant scores between all four biological replicates (Replica 1-4). All Pearson correlations are in the range 0.93 to 0.94.

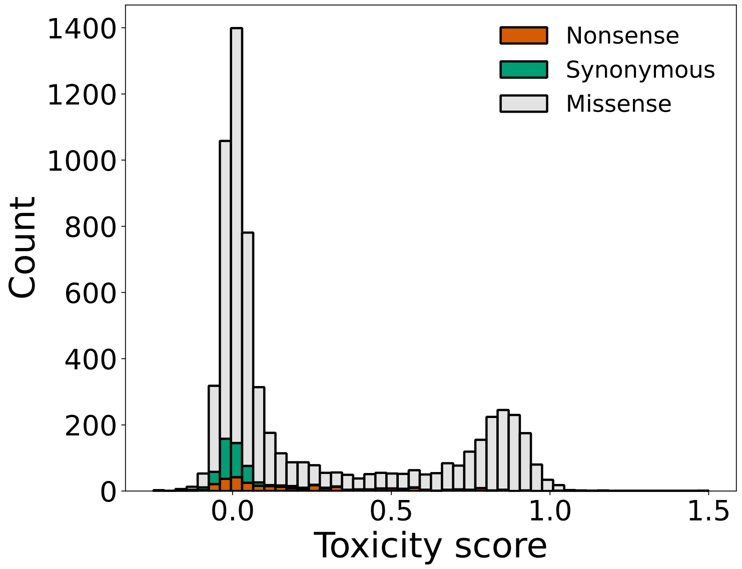

Supplementary Figure 13 – *Toxicity score distribution.*
Stacked histogram of toxicity scores for ASPA nonsense (red), synonymous (green) and missense (gray) variants. The missense variant toxicity score distribution is bimodal with a large peak around 0 originating from non-toxic variants and a smaller peak of toxic variants with toxicity scores close to 1. Note that while most nonsense variants are of low abundance (Fig.2), only a few of these appear toxic.

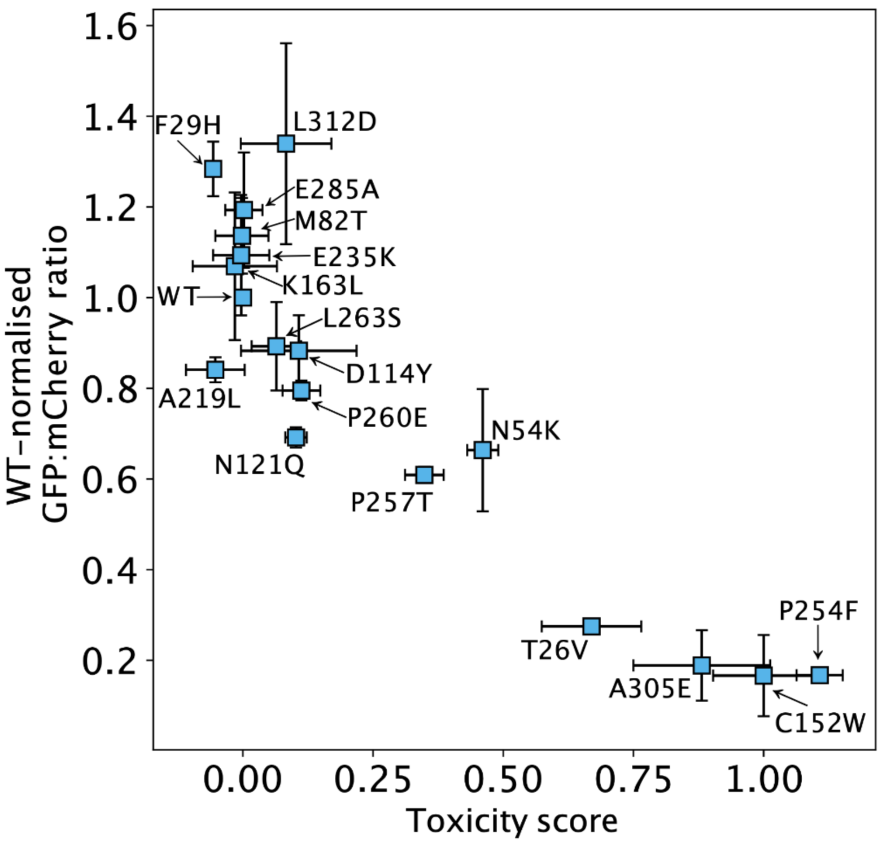

Supplementary Figure 14 –*ASPA toxicity scores and abundance determined in low throughput.*
To compare toxicity and abundance, the abundance of 17 ASPA variants and wild-type ASPA were analyzed one-by-one by flow cytometry in low throughout. The abundance scores determined in low-throughput (y-axis) correlate with the toxicity scores determined from the screen (x-axis). Error bars reflect the standard deviation (n = 3 independent experiments).

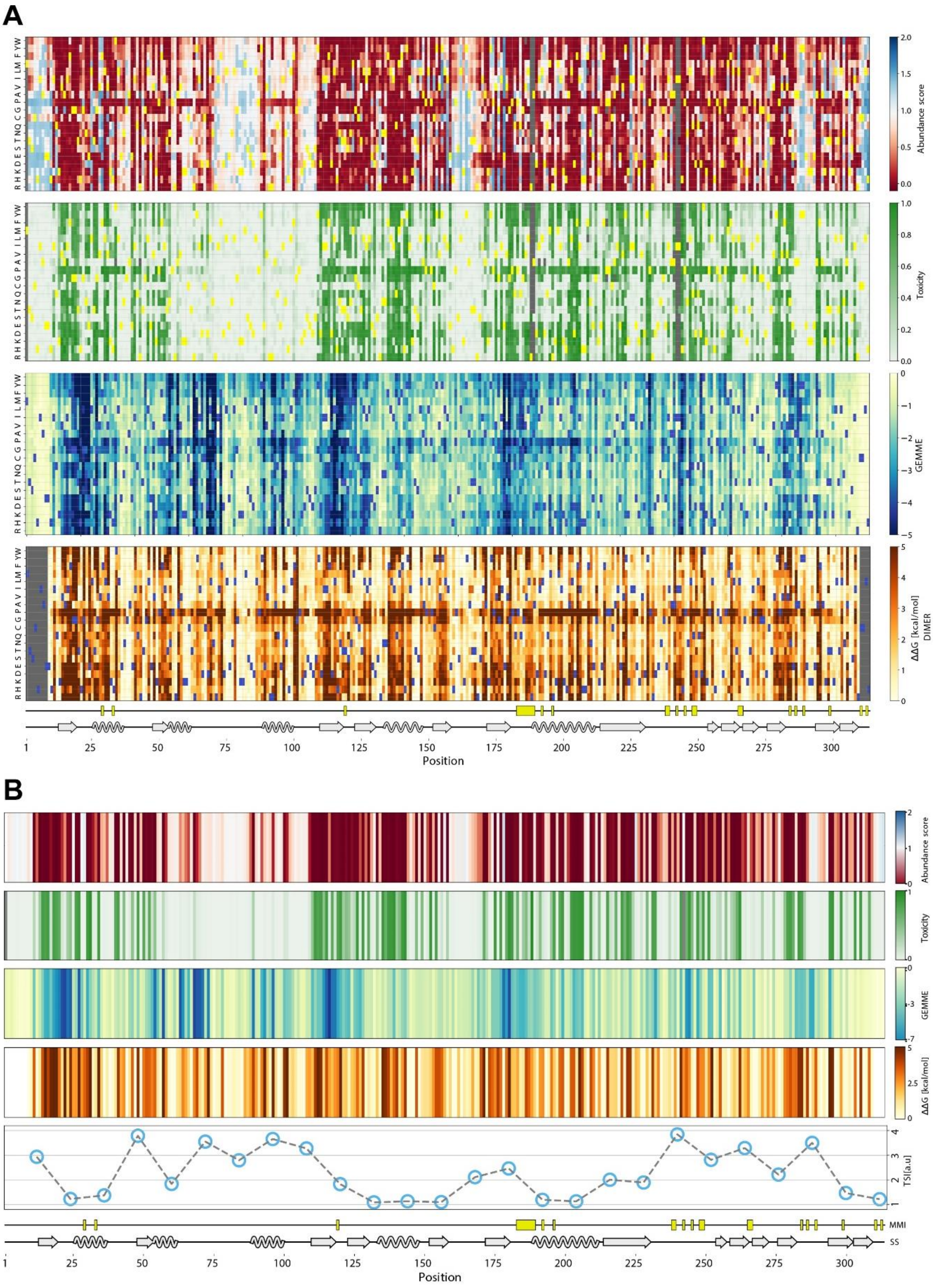

Supplementary Figure 15 – *Comparisons of the mutational maps.*

(A) Side-by-side comparisons of the ASPA abundance map, the toxicity map, the GEMME conservation scores and Rosetta predicted change in thermodynamic stability (ΔΔG) for the ASPA dimer. The secondary structure (SS) elements and monomer-monomer interface (MMI) positions are included for comparison. Scatter plots of these data are included in the main text (Fig. 7BCD). (B) Comparisons of the ASPA median abundances and toxicity per residue with the corresponding residue

median GEMME and median Rosetta stability predictions for the ASPA dimer. The tile stability index (TSI) is plotted for the ASPA tiles for comparison.

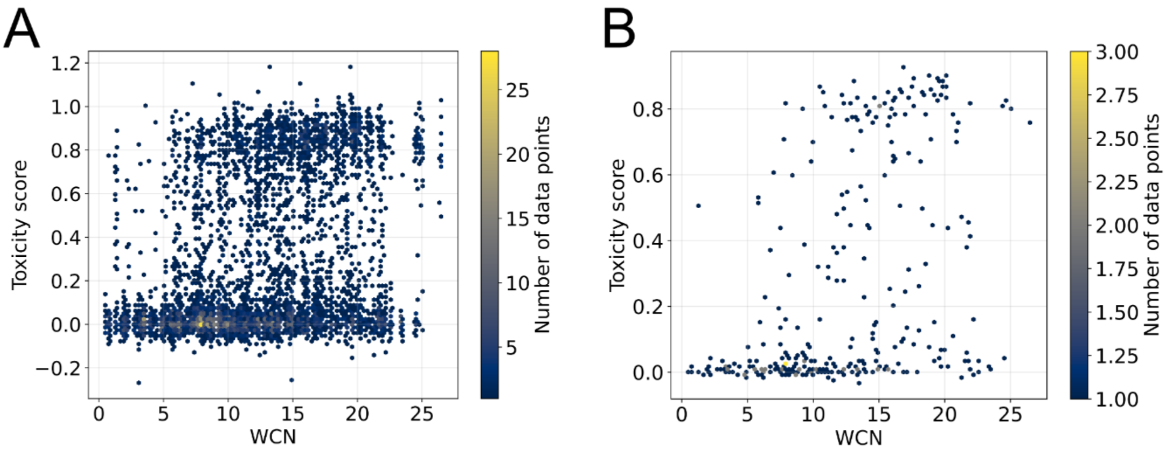

Supplementary Figure 16 – *Toxic variants are typically buried.*
Plots of the (A) toxicity scores and (B) median toxicity scores vs. the weighted contact number (WCN).

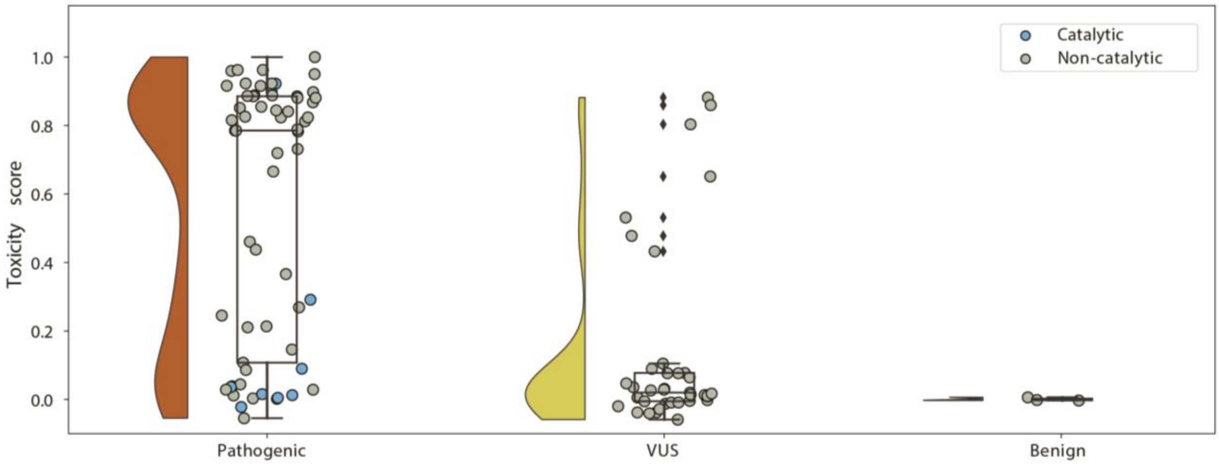

Supplementary Figure 17 – *Benign variants are not toxic*.
Comparisons of the toxicity scores for ASPA missense variants listed in the Source Data as pathogenic (red) (n = 61), variants of uncertain significance (VUS) (yellow) (n = 37) and benign (green) (n = 3) are shown as raincloud plots. Residues in or near the ASPA active site have been marked (blue). Note that many pathogenic and some VUS variants are toxic. Many of the non-toxic pathogenic variants are located near the active site (catalytic, blue). The box shows the quartiles of the dataset while the whiskers extend to show the rest of the distribution, except for points that are determined to be outliers (diamonds).

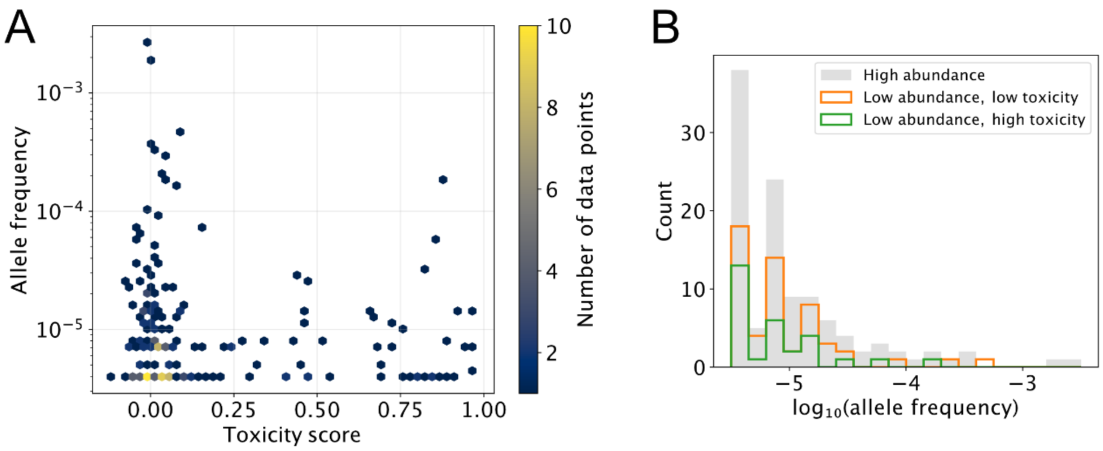

Supplementary Figure 18 – *Common variants are not toxic, and toxic variants are not depleted in the population.*

(A) Comparison of the ASPA toxicity scores with the ASPA allele frequencies reported in gnomAD. Note that ASPA variants that are common in the population are non-toxic, while some rare variants display high toxicity scores. (B) Allele frequency histograms for high abundance variants (grey), low abundance variants with low toxicity (orange) and low abundance variants with high toxicity (green). The three distributions all have a median $\log_{10}$(allele frequency) of -5.16 and have $\log_{10}$(allele frequency) averages of -4.97±0.054 (high abundance), -5.05±0.057 (low abundance, low toxicity) and -5.11±0.071 (low abundance, high toxicity). The toxic ASPA variants are thus not considerably depleted in the population compared to the non-toxic variants. All statistics are calculated on the log transformed data, and errors are standard deviations estimated using bootstrapping with 1000 bootstrap samples.

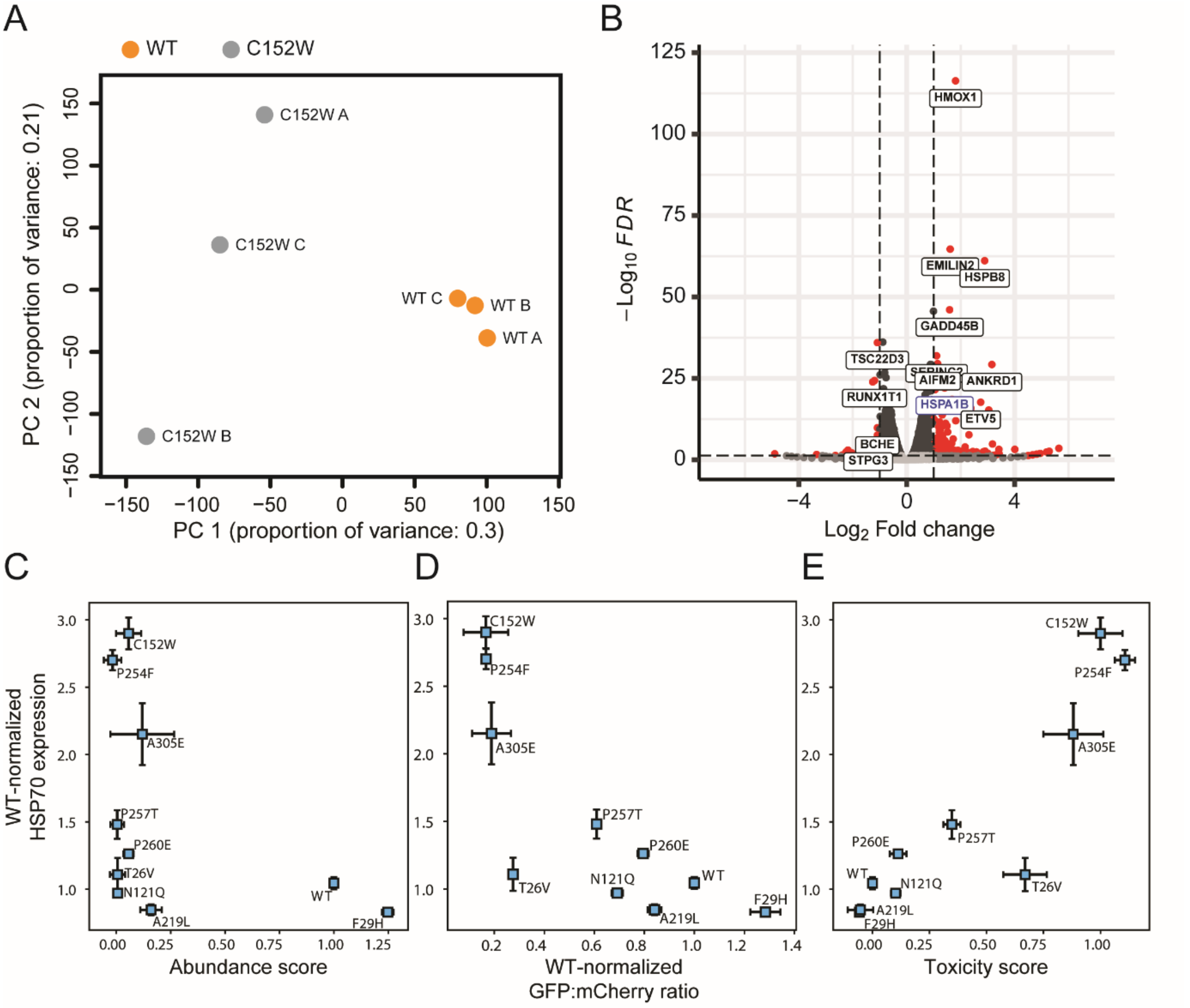

Supplementary Figure 19 – *Toxic variants activate a stress response leading to HSP70 induction.* (A) Principal component (PC) analysis (PCA) of RNA sequencing on cells expressing wild-type (WT) ASPA (orange) or C152W (grey). The three independent biological repeats (separate transfections) for each condition are marked A, B and C. (B) Differentially expressed genes between WT and C152W cells presented as a volcano plot. Genes, where the differential expression is statistically significant, are marked in red (FDR < 0.05 and |log2FC| > 1). (CDE) The expression of the HSP70 chaperone HSPA1A/HSPA1B was analyzed by qPCR in cells expressing the indicated ASPA variants (n=3 independent experiments) The error bars indicate the standard deviation. The HSP70 expression normalized to that in cells expressing WT ASPA is plotted (C) vs. the abundance score determined by VAMP-seq, (D) vs. the GFP/mCherry ratios determined in low throughput, and (E) vs. the toxicity score. The error bars reflect the standard deviation (n = 3 independent experiments).

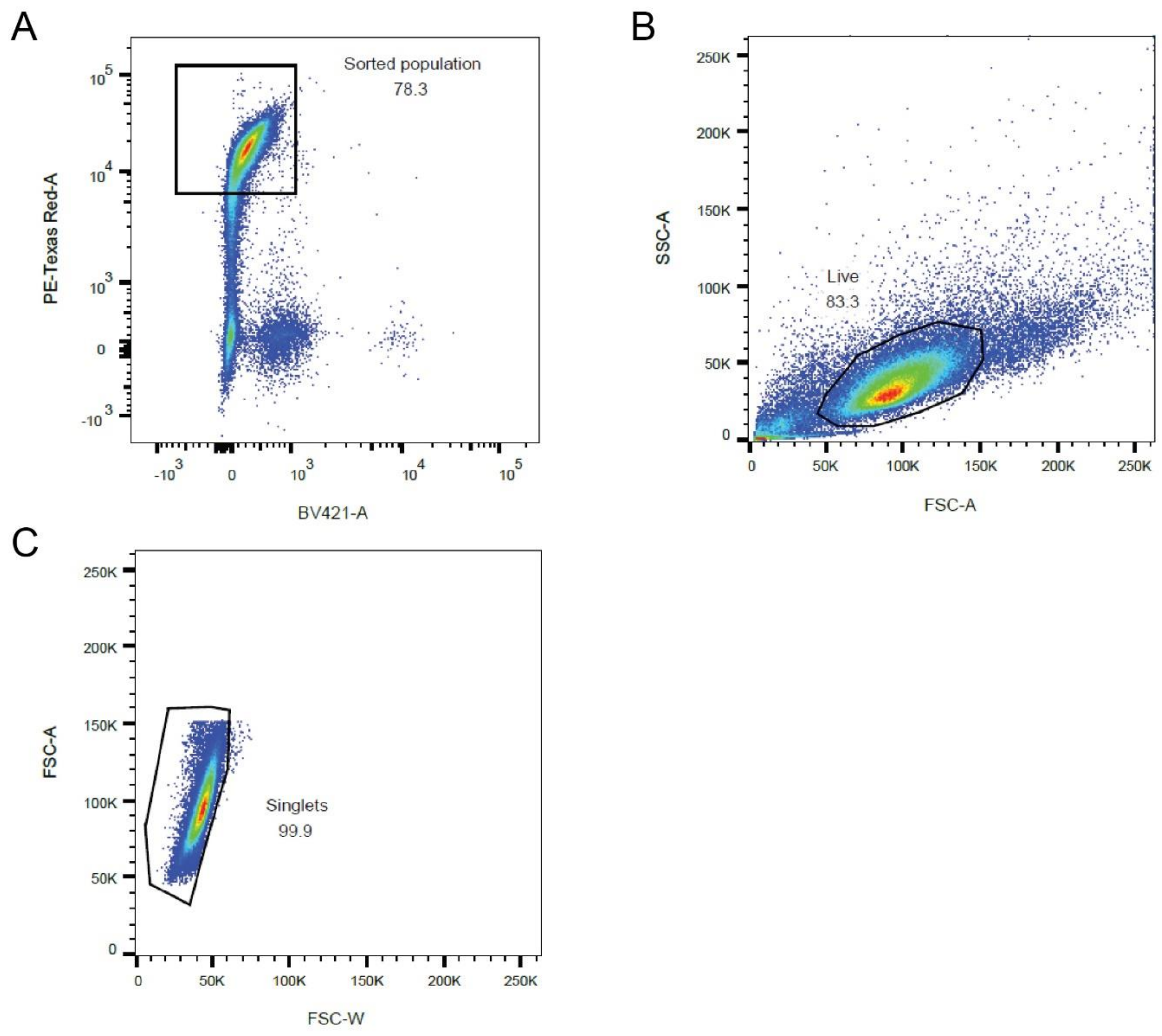

Supplementary Figure 20 – *Flow cytometry gating strategy.*

(A) The presented flow cytometry and FACS data were obtained by gating for the mCherry positive and BFP negative cells, corresponding to the boxed region. The shown data are for GFP-tagged ASPA library. In addition, back-gating, based on forward and side scattering, was applied to select for (B) live cells (the shown data are for GFP-tagged ASPA library) and (C) singlets (the shown data are for GFP-tagged ASPA library). The gating strategy applies to all the presented FACS data.
