# Supplementary material for: Deep mutational scanning reveals a correlation between degradation and toxicity of thousands of aspartoacylase variants
Source: Nat Commun. 2024 May 13;15:4026. doi: 10.1038/s41467-024-48481-0 (PMC11091098; doi:10.1038/s41467-024-48481-0)
Supplement: Supplementary file 3 — Reporting Summary [file 41467_2024_48481_MOESM3_ESM.pdf]

nature portfolio

Corresponding author(s): Douglas Fowler
Kresten Lindorff-Larsen
Rasmus Hartmann-Petersen

Last updated by author(s): April 24th 2024

Reporting Summary

Nature Portfolio wishes to improve the reproducibility of the work that we publish. This form provides structure for consistency and transparency in reporting. For further information on Nature Portfolio policies, see our Editorial Policies and the Editorial Policy Checklist.

Statistics

For all statistical analyses, confirm that the following items are present in the figure legend, table legend, main text, or Methods section.

n/a	Confirmed	
☐	☒	The exact sample size (*n*) for each experimental group/condition, given as a discrete number and unit of measurement
☐	☒	A statement on whether measurements were taken from distinct samples or whether the same sample was measured repeatedly
☒	☐	The statistical test(s) used AND whether they are one- or two-sided<br>*Only common tests should be described solely by name; describe more complex techniques in the Methods section.*
☒	☐	A description of all covariates tested
☒	☐	A description of any assumptions or corrections, such as tests of normality and adjustment for multiple comparisons
☐	☒	A full description of the statistical parameters including central tendency (e.g. means) or other basic estimates (e.g. regression coefficient) AND variation (e.g. standard deviation) or associated estimates of uncertainty (e.g. confidence intervals)
☒	☐	For null hypothesis testing, the test statistic (e.g. *F*, *t*, *r*) with confidence intervals, effect sizes, degrees of freedom and *P* value noted<br>*Give P values as exact values whenever suitable.*
☒	☐	For Bayesian analysis, information on the choice of priors and Markov chain Monte Carlo settings
☒	☐	For hierarchical and complex designs, identification of the appropriate level for tests and full reporting of outcomes
☐	☒	Estimates of effect sizes (e.g. Cohen's *d*, Pearson's *r*), indicating how they were calculated

Our web collection on statistics for biologists contains articles on many of the points above.

Software and code

Policy information about availability of computer code

Data collection	PacBio reads were filtered for reads with less than ten CSS passes using samtools version 1.16 and aligned to the barcode-GFP-ASPA construct using BWA version 0.7.17. The barcode and ASPA sequences were extracted using cutadapt version 3.2, see pacbio/pacbio_align.sh available on GitHub. Reads containing ten or more DNA substitutions or any indels were filtered out. For 1,436 barcodes (1%), multiple ASPA variants mapped to the same barcode but with the majority mapping to a dominant variant, on average 89% of reads of that barcode, which was then used. Illumina sequencing reads were converted to fastq and de-multiplexed using Basespace (Illumina). For RNA sequencing, quality control of sequence reads was done using the tools "FastQC" v0.11.7, "RSeQC" v2.6.4 99 and "fastq_screen" v0.11.4. Low-quality bases and the first 12 bases and reads shorter than 25 nt were removed with "Trimmomatic" v0.39. Reads were mapped using "STAR" v2.7.3. The "featureCounts" function of the "Rsubread" R package v2.2.6 was used to quantify reads in exons. The Gencode v38 comprehensive gene annotation was used to assign reads to genes. The "edgeR" v3.30.6 software was used to perform a differential expression analysis.
Data analysis	Illumina reads from the abundance and toxicity screens were cleaned for adapters using cutadapt 95 and paired-end reads were joined using fastq-join from ea-utils, see illumina/call_zerotol_paired.sh available on GitHub. Only barcodes with an exact match to the barcode map were counted, see illumina/merge_counts.r. Read counts of barcodes were merged for amino acid variants and the technical replicates of each FACS bin (abundance) or time point (toxicity) and normalized to frequencies without pseudo counts. After merging, a score was calculated for variants with 20 or more reads observed per replica. Similar to the above, the tile reads were cleaned for adapters sequences using cutadapt and paired end reads were joined using fastq-join from ea-utils. Only barcodes with an exact match to the barcode map were counted. If tiles from the Odds, Evens or CT libraries were observed in a sorting of a different library, these were assumed to be non-sorted contaminants and ignored. Technical replicates of each FACS bin were merged and normalized to frequencies without pseudo counts. For each library, biological and FACS replicas, a tile stability index (TSI) was calculated.<br>All data and software generated for this article is available on GitHub: https://github.com/KULL-Centre/_2023_Groenbaek-Thygesen_ASPA_MAVE (DOI:10.5281/zenodo.8382504).

Additional code that was used include:
Rosetta (GitHub SHA1 99d33ec59ce9fcecc5e4f3800c778a54afdf8504)
GEMME v1.0

For manuscripts utilizing custom algorithms or software that are central to the research but not yet described in published literature, software must be made available to editors and reviewers. We strongly encourage code deposition in a community repository (e.g. GitHub). See the Nature Portfolio guidelines for submitting code & software for further information.

Data

Policy information about availability of data

All manuscripts must include a data availability statement. This statement should provide the following information, where applicable:

- Accession codes, unique identifiers, or web links for publicly available datasets
- A description of any restrictions on data availability
- For clinical datasets or third party data, please ensure that the statement adheres to our policy

All data generated for this article is available on GitHub: https://github.com/KULL-Centre/_2023_Groenbaek-Thygesen_ASPA_MAVE (DOI:10.5281/zenodo.8382504). The DNA sequencing data have been deposited at the Gene Expression Omnibus (GEO), accession code: GSE254639 (https://www.ncbi.nlm.nih.gov/geo/query/acc.cgi?acc=GSE254639). Abundance and toxicity scores are also deposited at MaveDB (https: //www.mavedb.org) under accession number urn:mavedb:00000657-a. Sequencing reads for the abundance and toxicity scores are available at https: //doi.org/10.17894/ucph.3e05fe3a-4d7e-4d70-9056-18ed999e7e1e. The RNA seq. data have been uploaded to Gene Expression Omnibus (GEO): https: //www.ncbi.nlm.nih.gov/geo/ (accession number: GSE232399; samples GSM7329952-57).
The processed data are available in the source data file provided with this paper.

Research involving human participants, their data, or biological material

Policy information about studies with human participants or human data. See also policy information about sex, gender (identity/presentation), and sexual orientation and race, ethnicity and racism.

Reporting on sex and gender	Not applicable.
Reporting on race, ethnicity, or other socially relevant groupings	Not applicable.
Population characteristics	Not applicable.
Recruitment	Not applicable.
Ethics oversight	Not applicable.

Note that full information on the approval of the study protocol must also be provided in the manuscript.

Field-specific reporting

Please select the one below that is the best fit for your research. If you are not sure, read the appropriate sections before making your selection.

[x] Life sciences [ ] Behavioural & social sciences [ ] Ecological, evolutionary & environmental sciences

For a reference copy of the document with all sections, see nature.com/documents/nr-reporting-summary-flat.pdf

Life sciences study design

All studies must disclose on these points even when the disclosure is negative.

Sample size	No sample size calculations were performed. Sample sizes of at least 100-fold library coverage were selected (i.e. 6260 variants x 100 = 626,000) as this should be sufficient to minimize the risk of losing variants. Thus, to ensure reproducibility and full library coverage: The VAMP seq. screening was performed 11 times in total: 4 biological repeats (separate library transfections and selections) Run1-4, with 2-3 technical repeats of the sorting. The toxicity screen was performed with 4 biological repeats (separate library transfections and selections). At least 100-fold coverage of library complexity was maintained throughout (i.e. 6260 variants x 100 = 626,000). The tile sequencing was performed with 3 biological repeats (separate transfections and selections), each with 2 technical repeats of the sorting. We aimed for 1000-fold coverage. The correlations between repeats and the obtained coverage, indicate that the above precautions were sufficient.
Data exclusions	As described in the paper, PacBio reads comprising ten or more DNA substitutions or any indels were removed. Only barcodes with a perfect match to the barcode map were counted, as these are the only ones where we can be sure in the variant identification. These criteria were pre-established.
Replication	All attempts at replication were successful and are included in the paper. The VAMP seq. screening was performed 11 times in total: 4 biological repeats (separate library transfections and selections) Run1-4, with 2-3 technical repeats of the sorting. The toxicity screen was performed with 4 biological repeats (separate library transfections and selections). The tile sequencing was performed with 3 biological repeats (separate transfections and selections), each with 2 technical repeats of the sorting

Randomization	No samples were allocated into groups. This was not possible with the experimental design used.
Blinding	There were no group allocations. Blinding was unnecessary as the identity of the variants was unknown during the experiment.

Reporting for specific materials, systems and methods

We require information from authors about some types of materials, experimental systems and methods used in many studies. Here, indicate whether each material, system or method listed is relevant to your study. If you are not sure if a list item applies to your research, read the appropriate section before selecting a response.

Materials & experimental systems

n/a	Involved in the study	
☐	☒	Antibodies
☐	☒	Eukaryotic cell lines
☒	☐	Palaeontology and archaeology
☒	☐	Animals and other organisms
☒	☐	Clinical data
☒	☐	Dual use research of concern
☒	☐	Plants

Methods

n/a	Involved in the study	
☒	☐	ChIP-seq
☐	☒	Flow cytometry
☒	☐	MRI-based neuroimaging

Antibodies

Antibodies used	Primary antibodies:<br>Rabbit anti-ASPA, Western blotting, Thermo Fisher Scientific, PA5-29180, diluted 1:1000<br>Mouse anti-beta-actin, Western blotting, Sigma Aldrich, A5441, diluted 1:1000<br>Rabbit anti-GAPDH, Werstern blotting, Cell Signaling Technology, 14C10, diluted 1:1000<br>Rat anti-GFP, Western blotting, Chromotek, 3H9, diluted 1:1000<br>Mouse anti-RFP, Western blotting, Chromotek, 6G6, diluted 1:1000<br>Secondary antibodies:<br>HRP-anti-rat IgG (Invitrogen, 31470), diluted 1:5000<br>HRP-anti-mouse IgG (Dako, P0260), diluted 1:5000<br>HRP-anti-rabbit IgG (Dako, P0448), diluted 1:5000
Validation	The anti-ASPA, GFP, RFP (mCherry) are validated in the manuscript (show no reaction in untransfected control lysates).<br>Information of the anti-beta-actin and anti-GAPDH that were used as loading controls are available at the company websites:<br>anti-GAPDH (https://www.cellsignal.com/products/primary-antibodies/gapdh-14c10-rabbit-mab/2118) validated reactivity on human protein by Western blotting.<br>anti-beta-actin (https://www.sigmaaldrich.com/DK/en/product/sigma/a5441) validated on human protein by Western blotting.

Eukaryotic cell lines

Policy information about cell lines and Sex and Gender in Research

Cell line source(s)	The HEK293T cells containing the landing pad are from: Matreyek et al. (2020) Nucleic Acids Res. 48:e1. doi: 10.1093/nar/gkz910.
Authentication	Authentication was regularly checked by selection with AP1903 and testing for expression of BFP from the landing pad in untransfected cells.
Mycoplasma contamination	The cells tested negative for mycoplasma.
Commonly misidentified lines (See ICLAC register)	None were used.

Plants

Seed stocks

Report on the source of all seed stocks or other plant material used. If applicable, state the seed stock centre and catalogue number. If plant specimens were collected from the field, describe the collection location, date and sampling procedures.

Novel plant genotypes

Describe the methods by which all novel plant genotypes were produced. This includes those generated by transgenic approaches, gene editing, chemical/radiation-based mutagenesis and hybridization. For transgenic lines, describe the transformation method, the number of independent lines analyzed and the generation upon which experiments were performed. For gene-edited lines, describe the editor used, the endogenous sequence targeted for editing, the targeting guide RNA sequence (if applicable) and how the editor was applied.

Authentication

Describe any authentication procedures for each seed stock used or novel genotype generated. Describe any experiments used to assess the effect of a mutation and, where applicable, how potential secondary effects (e.g. second site T-DNA insertions, mosiacism, off-target gene editing) were examined.

Flow Cytometry

Plots

Confirm that:

- [x] The axis labels state the marker and fluorochrome used (e.g. CD4-FITC).
- [x] The axis scales are clearly visible. Include numbers along axes only for bottom left plot of group (a 'group' is an analysis of identical markers).
- [x] All plots are contour plots with outliers or pseudocolor plots.
- [x] A numerical value for number of cells or percentage (with statistics) is provided.

Methodology

Sample preparation

Cells were dislodged by thorough trypsination, stored in 5% (v/v) bovine calf serum in PBS and filtered through a 35 micrometer nylon mesh.

Instrument

Cells were sorted on a BD Biosciences ARIA III instrument equipped with a 70 micrometer nozzle. The laser used for excitation of BFP was 405 nm, for GFP 488 nm and for mCherry 562 nm. The filters used were 442/46 for BFP, 530/30 for GFP and 615/20 for mCherry. For analytical flow cytometry, we used a BD FACSJazz instrument. BFP was excited at 405 nm, GFP and mCherry were excited by a 488 nm laser and a 561 nm laser, respectively. Live single cells were gated using forward and side scatter before successfully recombined cells were gated on BFP negativity and mCherry positivity. The filters were 450/50 for BFP, 530/40 for GFP and 610/20 for mCherry.

Software

FACSDiva
FlowJo v.10.9.0

Cell population abundance

The cells were sorted into 4 equally populated bins based on the GFP/mCherry ratios. All the sorted cells were used for DNA sequencing. The controls (WT and C152W) were located in the expected bins. The VAMP seq. data correlated with low throughput validation of individual variants.

Gating strategy

1) The population of cells was gated by using FSC-A and SSC-A (for live cells) and FSC-A and FSC-H (for single cells).
2) The recombinant cells were selected based on their lack of BFP, and expression of mCherry. The mCherry signal was at least 10 times higher than the median fluorescence value of negative or control cells, and the BFP fluorescence signal was at least 10 times lower than the median of the unrecombined BFP positive cells.
3) A histogram of the GFP:mCherry ratiometric parameter was established on the FACSDiva software and gates were set to separate the whole library into four equally populated bins based on the GFP:mCherry ratio. An example of the gating strategy is shown in Fig. 1F and supplementary Fig. 20.

- [x] Tick this box to confirm that a figure exemplifying the gating strategy is provided in the Supplementary Information.
